# Atypical low-frequency cortical encoding of speech identifies children with developmental dyslexia

**DOI:** 10.3389/fnhum.2024.1403677

**Published:** 2024-06-07

**Authors:** João Araújo, Benjamin D. Simons, Varghese Peter, Kanad Mandke, Marina Kalashnikova, Annabel Macfarlane, Fiona Gabrielczyk, Angela Wilson, Giovanni M. Di Liberto, Denis Burnham, Usha Goswami

**Affiliations:** ^1^Centre for Neuroscience in Education, Department of Psychology, University of Cambridge, Cambridge, United Kingdom; ^2^Department of Applied Mathematics and Theoretical Physics, Centre for Mathematical Sciences, Cambridge, United Kingdom; ^3^The Wellcome Trust/Cancer Research UK Gurdon Institute, University of Cambridge, Cambridge, United Kingdom; ^4^School of Health, University of the Sunshine Coast, Maroochydore, QLD, Australia; ^5^Basque Center on Cognition, Brain, and Language, San Sebastian, Spain; ^6^Ikerbasque, Basque Foundation for Science, Bilbao, Spain; ^7^ADAPT Centre, School of Computer Science and Statistics, Trinity College, The University of Dublin, Dublin, Ireland; ^8^Trinity College Institute of Neuroscience, Trinity College, The University of Dublin, Dublin, Ireland,; ^9^MARCS Institute for Brain, Behaviour, and Development, Western Sydney University, Sydney, NSW, Australia

**Keywords:** speech, developmental dyslexia, oscillations, unsupervised learning, supervised learning, classification, common spatial patterns

## Abstract

Slow cortical oscillations play a crucial role in processing the speech amplitude envelope, which is perceived atypically by children with developmental dyslexia. Here we use electroencephalography (EEG) recorded during natural speech listening to identify neural processing patterns involving slow oscillations that may characterize children with dyslexia. In a story listening paradigm, we find that atypical power dynamics and phase-amplitude coupling between delta and theta oscillations characterize dyslexic versus other child control groups (typically-developing controls, other language disorder controls). We further isolate EEG common spatial patterns (CSP) during speech listening across delta and theta oscillations that identify dyslexic children. A linear classifier using four delta-band CSP variables predicted dyslexia status (0.77 AUC). Crucially, these spatial patterns also identified children with dyslexia when applied to EEG measured during a rhythmic syllable processing task. This transfer effect (i.e., the ability to use neural features derived from a story listening task as input features to a classifier based on a rhythmic syllable task) is consistent with a core developmental deficit in neural processing of speech rhythm. The findings are suggestive of distinct atypical neurocognitive speech encoding mechanisms underlying dyslexia, which could be targeted by novel interventions.

## Introduction

Developmental Dyslexia affects around 7% of children in all languages, negatively impacting education and life chances (Lyon et al., 2003). Behavioral research shows that atypical linguistic processing lies at the heart of dyslexia (Stanovich, 1998; Snowling et al., 2000). Neural studies reveal that even as infants, individuals at family risk for dyslexia show both atypical auditory processing and atypical speech processing (EEG and MEG studies; Guttorm et al., 2001, 2003; Leppänen et al., 1999, 2002; van Leeuwen et al., 2006; van Zuijen et al., 2013; Mittag et al., 2021). While dyslexia is diagnosed primarily on the basis of difficulties in reading and spelling once schooling begins, at-risk children show atypical processing of phonology (the sound structure of speech) as preschoolers (Goswami, 2022a, for recent review). Crucially, these phonological difficulties cannot be attributed to lower intellectual ability, overt hearing impairment or lower-quality home learning environments (Scarborough, 1990; Lyytinen et al., 2006). Linguistically, recent neural research has shown across languages that dyslexia in children is characterized by impairments in processing the speech amplitude envelope, with associated difficulties in processing speech rhythm (Goswami, 2022a, for review). For example, cortical speech tracking of the amplitude envelope in both the delta (0.5 – 4 Hz) and theta (4 – 8 Hz) electrophysiological bands (thought to be critical for encoding syllabic and prosodic [speech rhythm] information) has been shown to be impaired in children with dyslexia who are learning English, French or Spanish, in both connected speech listening tasks and sentence repetition tasks (Molinaro et al., 2016; Power et al., 2016; Di Liberto et al., 2018; Destoky et al., 2020, 2022; Mandke et al., 2022; Keshavarzi et al., 2022a). The dyslexic brain also shows a different preferred phase in the delta band when listening to rhythmic syllable repetition (repetition of the syllable “ba” at a 2 Hz rate; Power et al., 2013; Keshavarzi et al., 2022b), and both cortical tracking and preferred phase measures are related to individual differencs in phonological awareness.

Theoretically, it has been hypothesized that the impairments in speech envelope processing found in children with dyslexia are related to atypical neural oscillatory responses: ‘Temporal Sampling’ (TS) theory (Goswami, 2011, 2015, 2022b). Linguistically, TS theory proposes that children with dyslexia are impaired at extracting prosodic information from the speech signal, which affects the development of phonological awareness at all linguistic levels (stressed syllables, syllables, onset-rimes, phonemes). The TS framework proposes that atypical oscillatory processing of amplitude modulation (AM) information at lower frequencies (<10 Hz, hence related to the EEG delta and theta frequency bands) is related to the speech rhythm impairments found in children with dyslexia. For example, children with dyslexia show impairments in perceiving syllable stress patterns, a fundamental component of speech rhythm carried by low-frequency AMs in the speech envelope (Goswami et al., 2010, 2013). Perceiving syllable stress depends in part on the accurate perception of amplitude rise times (ARTs), as stressed (strong) syllables have larger rise times (Greenberg, 2006). Accordingly, the speech rhythm impairments found in children with dyslexia may also be related to impaired perceptual and neural mechanisms of speech edge detection (ART detection, Lizarazu et al., 2021; Mandke et al., 2023). Speech edges or ARTs are known to play an automatic role in phase-resetting neural oscillations during speech encoding (Gross et al., 2013; Doelling et al., 2014). Consistent with an atypical speech edge processing account, a recent MEG study with dyslexic children found that neural responses in the delta and theta frequency bands during story listening became less atypical if amplitude rise times and delta-band AMs were enhanced by filtering natural speech (Mandke et al., 2023).

To date, however, no studies have identified neural mechanisms capable of distinguishing children with dyslexia from typically-developing children based on temporal parameters in naturalistic speech conditions. To remedy this omission, here we examined the relative magnitude and coupling of low-frequency neural oscillations –delta and theta, as identified by TS theory – during two different receptive speech tasks, story listening and rhythmic repetition of the syllable “ba.” Given the impairments in ART discrimination that are found in both infants at family risk for dyslexia (Kalashnikova et al., 2018) and children with dyslexia [see Goswami (2022a)], it could be expected that individuals with dyslexia have impaired neural phase-resetting mechanisms, which may contribute to the impairments documented in delta- and theta-band cortical speech tracking in different languages (English, Power et al., 2016, Di Liberto et al., 2018, Mandke et al., 2022, Keshavarzi et al., 2022a; French, Destoky et al., 2020, 2022; Spanish, Molinaro et al., 2016). Oscillatory cross-frequency coupling (CFC) is also known to be important in encoding the speech signal (Giraud and Poeppel, 2012; Gross et al., 2013). Accordingly, atypical CFC could also potentially identify individuals with dyslexia. Neurally, the primary auditory cortex is organized in phase-amplitude hierarchies, so that delta phase modulates theta phase and amplitude, and the theta phase controls gamma amplitude (Lakatos et al., 2005; Gross et al., 2013). Changes in low-frequency oscillatory coupling (namely delta and theta) have been reported both when stimulus rhythm is relevant to the task (Schroeder and Lakatos, 2009) and in long time windows of audio-visual stimulus integration (Schroeder et al., 2008). Therefore, studying the relative magnitude and coupling of delta and theta oscillations in children with and without dyslexia may identify sensory-neural deficits that can uniquely classify children with developmental dyslexia, offering novel biomarkers. As well as improving diagnosis, improved understanding of potential sensory-neural deficits may enable the development of novel interventions for children with dyslexia, such as BCIs (Brain-Computer Interfaces; see Araujo et al., 2024). Our first research question was thus whether it would be possible to identify different delta/theta dynamics in children with dyslexia during natural speech processing. Our second research question was whether different delta/theta dynamics would also occur during the simpler rhythmic speech processing task (“ba.ba.ba”).

Recent EEG studies using rhythmic auditory *non-speech* stimuli with dyslexic children have shown that it is possible to build dyslexia classifiers [white AM noise, see Ortiz et al. (2020) and Gallego-Molina et al., (2022). These prior classifier studies used several different types of features (time, frequency, fractal or CFC graph networks). They employed large and complex non-linear models, achieving good performance metrics (>80%). However, due to the inherent nature of these modeling approaches, the connection between model parameters for neural data and the underlying cognitive/linguistic processes is not transparent. By contrast, here we aimed to develop an interpretable EEG classifier that can distinguish reliably between children with dyslexia and typically-developing (TD) control children. Our third research question was whether distinct patterns of oscillatory dynamics during natural speech listening would be robust enough to build a classifier for dyslexia, and our fourth question was whether a reliable classifier could be based on the same neural features for natural connected speech and simplified rhythmic speech. By comparing spatial patterns of oscillatory activity from two different receptive speech tasks (natural speech and rhythmic speech), we also aimed to shed light on how the estimated biomarkers might relate to cognitive/linguistic processes, such as rhythm processing.

As well as employing TD controls, we compared the neural oscillatory responses hypothesized as core by TS theory to those exhibited by a small group of children presenting with a different linguistic disorder, Developmental Language Disorder (DLD). This group is conceptually important in order to identify potential neural features that are exclusive to dyslexia. We predicted that the relative amplification of theta versus delta responses and delta-theta cross-frequency coupling might also be altered in children with dyslexia compared to DLD children. For example, TS-theory driven modeling of the speech amplitude envelope shows that delta-theta AM phase alignment underpins speech rhythm perception, accordingly delta-theta coupling could be atypical in children with dyslexia (Leong et al., 2014; Leong and Goswami, 2015; see research questions 1 and 2). We also hypothesized that delta and theta oscillatory responses to connected speech might provide sufficient information to classify neural patterns that identify dyslexia at the single trial level (see research question 3). Finally, we investigated how specific or universal these discriminatory patterns might be to natural language versus general rhythmic speech processing (see research question 4). We were able to do this using EEG recorded during a rhythmic syllable repetition task collected with different dyslexic and TD children (but no DLD children). By training a classifier on the EEG from rhythmic syllable listening using discriminatory features derived from story listening, we may find a generalization of features for classification. If this were the case, our modeling would suggest that classification of dyslexia is related to atypical neural processing of linguistic rhythm, rather than semantic or syntactic processing. As the aim of the current modeling was to distinguish neural characteristics of the dyslexic brain, the typically-developing children in the two samples covered a range of ages, language levels and reading levels.

## Materials and methods

### Participants

We analsyed EEG collected from two different groups of children drawn from community samples, 65 Australian English children who received the story listening paradigm and 48 British English children who received the rhythmic syllable repetition paradigm (see Experimental Tasks below). The Australian sample included typically-developing children (with similar age as dyslexic children – CA – or younger children with similar reading level as dyslexic children – RL), dyslexic children and a small group of children with Developmental Language Disorder (DLD). The latter were included as a control group that, while not typically-developing, present with a language disorder distinct from dyslexia (hence would be expected to show different neural features to children with dyslexia). Australian children received a range of neuropsychological and cognitive tests, see Table 1. To be included, participants had to have no reported history of hearing difficulties, non-verbal IQ scores within the normal range (85 and above) on the Kaufman Brief Intelligence Test (KBIT, Hildman et al., 1993). To qualify as dyslexic, children had to score at least 1 SD below the norm of 100 for single word or nonword reading measures on the Test of Word Reading Efficiency (84 and below, Torgesen et al., 1999), and show average scores (less than 1 SD from the norm of 100, so 85 or above) on at least one of the measures of language development, the TROG (Test of Receptive Oral Grammar, Bishop, 2003), CELF (Clinical Evaluation of Language Fundamentals, Wiig et al., 2000) or WIAT Vocabulary (Wechsler, 2009). Children also received the CTOPP (Comprehensive Test of Phonological Processing, Wagner et al., 1999) as a measure of phonological awareness, this test requires the oral blending or elision of words, syllables and other phonological units. To qualify as DLD, children had to receive standard scores at least 1 SD below the norm of 100 for the TROG, WIAT and CELF measures, and an average score (less than 1 SD from the norm of 100) on the reading measures. Please note that the vocabulary test was changed during the Australian project from the CELF vocabulary scale to the WIAT vocabulary scale. Fewer DLD children (*N* = 7) were in the Australian sample than dyslexic children (*N* = 16). No participants with ADHD were included. After the pre-processing pipeline, 2 typically-developing participants were excluded due to noisy EEG measurements, hence data from a total of 63 Australian participants were analyzed.

**Table 1:** Details of the Australian sample.

	**TD controls**		**DYSLEXIC** *n* = 16 [6F]	**DLD** *n* = 7 [2F]
	RL *n* = 13 [7F]	CA *n* = 27 [10F]		
Age (months)	83.7 (6.0)	113.6 (13.1)	114.1 (18.5)	95.0 (15.3)
PH_AW^a^	106.4 (10.0)	103.7 (10.5)	86.0 (11.4)	91.9 (8.0)
TOWRE_W^b^	100.9 (15.6)	102.6 (14.2)	77.3 (11.1)	94.4 (6.9)
TOWRE_NW^c^	99.2 (14.7)	102.5 (13.3)	78.2 (8.2)	94.7 (12.7)
TROG^d^	106.5 (6.7)	106.0 (8.6)	101.1 (9.5)	81.7 (16.1)
WIAT_VOCAB^e^	107.5 (11.8)	106.7 (11.3)	102.4 (10.8)	90.1 (12.4)
CELF_SENTENCES^f^	11.2 (2.7)	11.8 (2.3)	9.4 (2.8)	7.3 (2.3)
NVIQ^g^	111.6 (11.4)	114.4 (9.0)	104.9 (9.3)	99.0 (12.2)

TD, typically developing; RL, reading level matched; CA, chronological age matched; DLD, developmental language disorder. ^a^Comprehensive test of phonological processing. ^b^Test of word reading efficiency: words. ^c^Test of word reading efficiency: non-words. ^d^Test for reception of grammar. ^e^WIAT vocabulary. ^f^Clinical evaluation of language fundamentals- recalling sentences (*M* = 10, SD = 3). ^g^Non-verbal IQ: Kaufman brief intelligence test-matrices. Number of female participants (F) in squared brackets. Standard deviations in parentheses.

The British English dataset included data from 48 children comprising two groups: typically-developing children (chronological age controls) and dyslexic children. Typically-developing versus dyslexia status was also ascertained by neuropsychological and cognitive testing (see Table 2). Participants had full scale IQs (FSIQ) in the normal range (85 and above) as estimated from four subtests of the Wechsler Intelligence Scale for Children (WISC, Wechsler, 2016): similarities, vocabulary, block design and matrix reasoning (Aubry and Bourdin, 2018), and had also passed a short hearing screen, based on the ability to hear pure tones of various frequencies presented at 20 dB hearing Level (HL). To qualify as dyslexic, children had to score at least 1 SD (15 standard points) below the standard score of 100 on at least two of 4 measures of single word or nonword reading and spelling (scoring 84 or less on the British Ability Scales, BAS, Elliott et al., 1996, and TOWRE). Children also received the Phonological Awareness Battery (PhAB, Frederickson et al., 1997) rhyming test as a measure of phonological awareness.

**Table 2:** Details of the British sample.

	**TD age-matched controls** *n* = 21 [6F]	**DYSLEXIC** *n* = 27 [13F]
**Age (months)**	109.1 (5.4)	109.3 (6.8)
**PHAB_R** ^a^	102.6 (5.7)	90.9 (11.5)
**TOWRE_W** ^b^	101.1 (7.7)	79.4 (13.3)
**TOWRE_NW** ^c^	98.0 (8.6)	76.9 (9.3)
**BPVS** ^d^	103.3 (11)	103.7 (11.6)
**BAS_R** ^e^	99.5 (6.2)	80.3 (7.4)
**BAS_S** ^f^	97.0 (6.1)	78.6 (6.1)
**FSIQ** ^g^	104.1 (10.8)	101.7 (9.9)

TD, Typically developing. ^a^Phonological assessment battery: rhyme awareness. ^b^Test of word reading efficiency: words. ^c^Test of word reading efficiency: non-words. ^d^British picture vocabulary scale. ^e^British ability scales: reading. ^f^British ability scales: spelling. ^g^Full Scale IQ. For all tests *M* = 100; SD = 15. Number of female participants [F] in squared brackets. Standard deviations in parentheses.

### Experimental tasks

**Figure 1:**
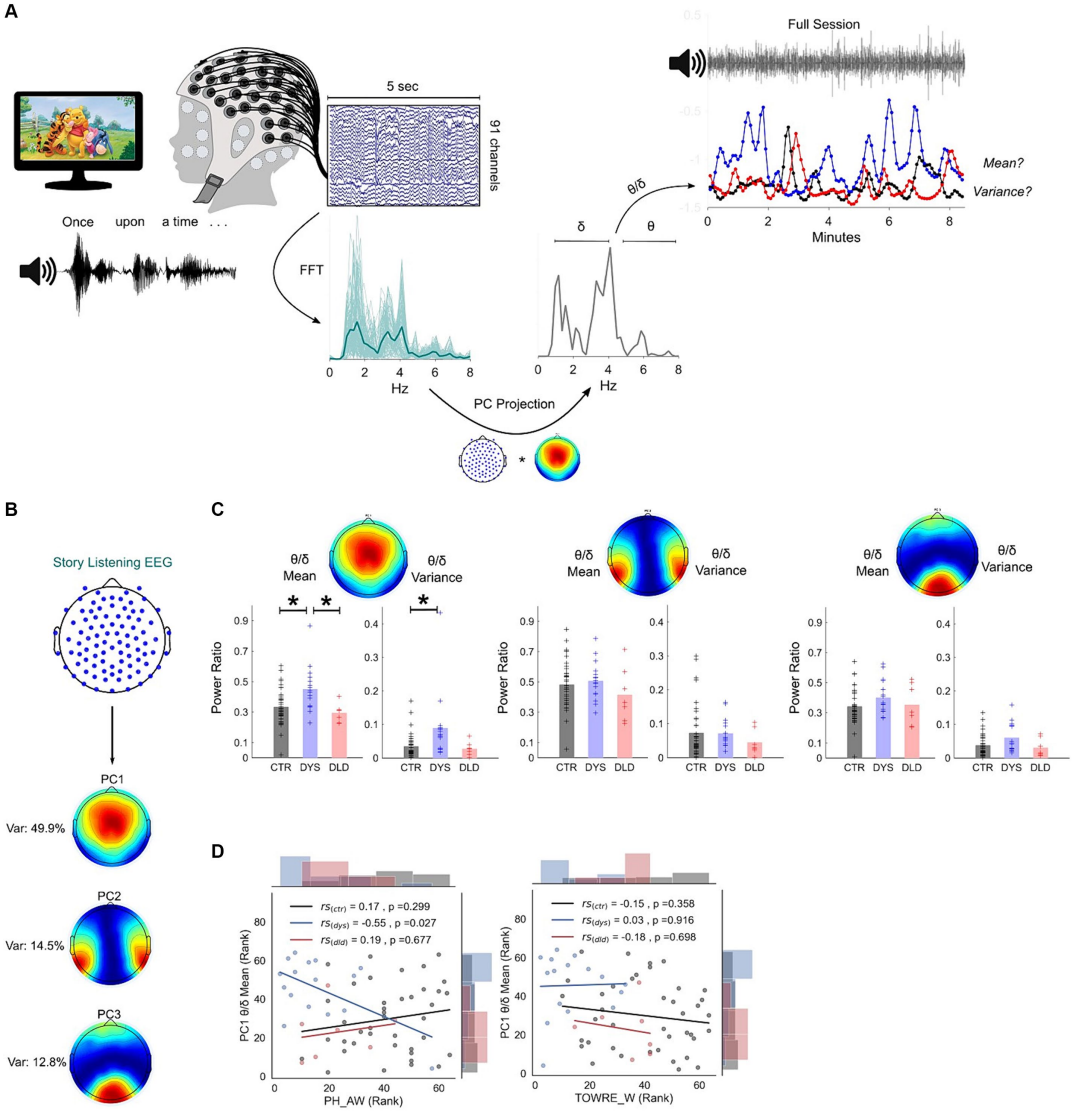
Dyslexic children show different theta/delta oscillatory power ratios during natural speech processing. **(A)** Story listening paradigm (Winnie-the-Pooh) and pseudo-online analysis pipeline (see Methods). Dimension reduction analysis was applied to EEG data using principal component analysis. EEG projection to principal component (PC) 1 is used for illustration. Examples of story sessions from a dyslexic (blue), DLD (red) and a typically-developing child (black) are shown at top right. **(B)** Map of channel weights of the three most important principal components: weights and respective percentage of variance explained. **(C)** Theta/delta ratio mean and variance across all three PCs. Atypically high mean and variance is present for dyslexics on PC1 (CTR – typically-developing children, DYS – dyslexic children, DLD – DLD children). * *p* < 0.05 (Bonferroni-corrected). **(D)** Spearman correlations and uncorrected *p*-values between PC1 theta/delta and phonological awareness (leftmost panel) and reading (timed single word reading test, right panel).

Two EEG datasets generated in prior studies by our group were used for the modeling (Di Liberto et al., 2018; Keshavarzi et al., 2022b). The first data set was collected during a story-listening task (see Di Liberto et al., 2018), and the second data set was collected during a rhythmic syllable repetition task [listening to the syllable “ba” repeated every 500 msec, see Keshavarzi et al. (2022b)]. In each case, cortical activity was recorded using the scalp potentials measured by non-invasive EEG. In the story listening paradigm, participants were presented with an audio-story for 9 min read by a female Australian English speaker while EEG was recorded (see Figure 1A). The stimulus was presented monophonically at a sampling rate of 44,100 Hz using loudspeakers in a silent room. Participants also watched a cartoon corresponding to the story (Winnie the Pooh), but the visual input was not synchronized to the detailed temporal events coming from the auditory modality (i.e., to the speech). EEG was recorded using 129-channel Hydrocel Geodesic Sensor Net (HCGSN), NetAmps 300 amplifier and NetStation 4.5.7 software (*EGI* Inc.). The sampling rate of this system was 1 kHz and channel impedances were always kept below 50 kΩ throughout the session.

The second paradigm was a rhythmic entrainment task (Keshavarzi et al., 2022b). Children listened to rhythmic speech comprising multiple repetitions of the syllable “ba” at a 2 Hz rate. In common with the story-listening task, the auditory targets were presented at 44100 Hz, this time via in-earphones. Synchronized visual information (a “talking head” providing articulatory cues onsetting 68 ms before the syllable onset) was also presented (synchronized audio-visual task). Participants were instructed to concentrate their gaze on the lips of the talking head to prepare for every new trial. In each trial, the syllable “ba” was presented 14 times and the child was instructed to press a key on the keyboard if they detected any syllable that violated the uniform 2 Hz rhythm. Feedback was presented after each 14-item trial. The full experimental session consisted of 90 trials divided into 3 blocks of 30. Each block comprised 25 trials where a rhythmic violation would occur randomly between the 9^th^ and 11^th^ syllable and 5 catch (i.e., absence of rhythmic violation) trials. For the rhythmic violation trials, the degree to which the violator was out of sync changed with each child’s performance on the task to optimize their level of engagement. This was achieved by a three-down one-up staircase procedure – if a child correctly identified 3 violations in a row, the stimulus onset asynchrony would be reduced 16.67 ms on the following non-catch trial and if a violator was not detected, the deviation would be increased 16.67 ms (Keshavarzi et al., 2022b). Similar to the story-listening set-up, a 129-channel Hydrocel Geodesic Sensor Net was used to record EEG scalp potentials during the task. The sampling rate was 500 Hz and electrode impedances were kept below 50 kΩ.

### EEG signal pre-processing

The pre-processing pipeline was similar for both the story listening and rhythmic syllable repetition data to ensure analysis consistency and maximize the validity of cross-set comparisons. The EEG signal was first bandpass-filtered between 1 and 25 Hz using a 4^th^ order Butterworth filter to remove very low-frequency and high-frequency noise (including power line noise). A zero-phase filtering method (backwards and forwards filtering – using the *filtfilt* function in MATLAB) was used to prevent phase shifting and reconstruct the original properties of the signal of interest as much as possible. Data were subsequently re-referenced to mean mastoids. Since one of the objectives of this study was to classify signals in a window length suited for Brain-Computer Interface (BCI) applications – whether for potential diagnosis or operant learning interventions – data was epoched to 5-s windows. The epoching of the story-listening data in short windows not only provides a way to evaluate the behavior of any EEG signal metric across the session but also allows for the comparison with the rhythmic syllable trials. Specifically, this epoching consisted in the extraction of consecutive non-overlapping EEG data windows representing 5 s of data – a procedure that was similar for both the story listening and the rhythmic syllable task, allowing cross-task comparison. To further remove noise sources like blinks or EMG activity, for each trial, channels with voltages over the absolute value of 100 μV were considered noisy and were interpolated using the spline method (EEGLAB; Delorme and Makeig, 2004). If over a third of the total number of channels were considered noisy, the trial was excluded. As in Di Liberto et al. (2018), data were downsampled to 100 Hz to reduce processing time and memory requirements, and EEG electrodes positioned at the jaw, mastoids, and forehead were removed from the analysis (91 channels total, see Figure 1B). After the pre-processing pipeline, each participant kept on average 88.63 epochs (st.d = 29.3) for the story listening task, and 45.23 epochs (st.d = 18.61) for the rhythmic syllable task.

### Deriving unsupervised spatial filters using principal components analysis

Given the absence of a specific hypothesis on which channel or channel ensembles would be the most appropriate to pinpoint group differences, a data-driven (albeit group-blind) way of estimating adequate spatial filters was derived. Principal Components Analysis (PCA) is used in this work to (1) allow dimensionality reduction of a 91-dimensional space of channels and in the process (2) create relevant spatial ensembles of channels that represent distinct (uncorrelated) sources of cortical activity. This ensemble analysis aims to create a more meaningful basis for analysing the whole-brain EEG signal than just relying on multiple single-channel analyses. In general, PCA will find the set of vectors *A* that project the original data *X* into a new space explaining the maximum original uncorrelated variance in the least number of vectors possible. This is one of the bottom-up methods for deriving spatial filters for EEG in an unsupervised manner (i.e., without looking at group labeled data). PCA can be conceptualized as an eigenvalue problem:


(1) $$A^{T}\Sigma A=\Lambda s.t.A^{T}A=I$$

where $\Sigma =X^{T}X.$ In practice, the matrix *A* was estimated using Singular Value Decomposition (*svd* function in MATLAB) and consists of a set of eigenvectors sorted by their eigenvalues (diagonal elements of matrix Λ ). The data *X* consisted of the concatenation of standardized *t* epoch segments X_t_ ∈ ℝ^dxN^ (where *d* is the number of channels and N is the number of datapoints on an epoch) along the second dimension so that X ∈ ℝ^dxM^ with M=total number of trials∗N . The relative channel weights for each PC were determined simply as the square of their original coefficient for each a∈A , since $\sum a^{2}=1$ . Initially, to decide on how many principal components to retain (i.e., the total new dimensionality of the data), a threshold of 70% variance explained was used. 3 PCs were found to be sufficient in both the story listening and the rhythmic syllable repetition EEG data (Figure 1B). Additionally, a further analysis of the scree plots (Figures S1a, S2a) revealed minimal increases of variance explained after PC3 which would usually explain 10 times as much variance as the next best PC on both datasets. A quick look at the channel weights of the first 10 PCs (Figures S1b, S2b) suggests the first 3 PCs for the story listening and rhythmic syllable repetition EEG datasets are less scattered and more biologically plausible when compared to the other 7 PCs. Taken together, this evidence provided additional confidence in choosing 3 as the optimal number of PCs to retain.

### Principal component band power ratio

To calculate the band power ratio for every PC band, Welch’s method was used first to estimate the broadband power spectral density of each epoch projected in each PC. A single Hanning window covering the epoch’s full length (500 datapoints at a sampling frequency of 100 Hz) was used. In practice, this was calculated using MATLAB’s Welch method implementation in the *pwelch* function. Each band power was calculated by averaging the discrete Fourier transform points belonging to each frequency band interval (delta: 1-4 Hz theta: 4.5-8 Hz). The theta/delta band ratio was calculated by dividing the averaged band power of theta and delta for each epoch. The band ratio metrics for each subject were calculated by taking the low order statistics (mean and variance) of their epochs’ theta/delta ratios across the session.

### Phase-amplitude coupling (PAC)

To estimate phase and amplitude for each epoch on the PAC analysis we relied on a recently published time-frequency approach that does not rely on bandpass filtering (Aviyente et al., 2011; Munia and Aviyente, 2019). Indeed, bandpass filtering artefacts, such as approximating the filtered signal to a sinusoid, especially in small bandwidth and high order filters, can be problematic for PAC estimation. Note only does the time-frequency approach used here solve this problem, but it also has interesting properties such as high frequency resolution and is more robust to noise, different data lengths and sampling rates (Munia and Aviyente, 2019). The method relies on the complex time-frequency distribution based on the interaction of energy at frequency *f* at a given time *t* known as Reduced Interference Distribution (RID) – Rihaczek distribution. The RID-Rihaczek distribution is a modified version of the Rihaczek distribution that uses the Choi-Williams kernel to filter out the cross-terms:


(2) $$C\left(t,f\right)=\int \int \exp\left(-\frac{{\left(\theta \tau \right)}^{2}}{\sigma }\right)\exp\left(j\frac{\theta \tau }{\sigma }\right)A\left(\theta ,\tau \right)e^{-j\left(\theta t+2\pi f\tau \right)}d\tau d\theta ,$$

where $\exp\left(-\frac{{\left(\theta \tau \right)}^{2}}{\sigma }\right)$ is the Choi-Williams kernel, $\exp\left(j\frac{\theta \tau }{\sigma }\right)$ is the kernel function of the Rihaczek distribution and A(θ,τ) is the ambiguity function of the given signal x(t) . This distribution belongs to Cohen’s class of distributions, reflecting the time-varying energy and phase of the signal. For an analytic signal $x\left(t\right)=A\left(t\right)e^{j\phi \left(t\right)}$ , with Fourier transform $X\left(f\right)=B\left(f\right)e^{j\theta \left(f\right)}$ , the instantaneous amplitude (for higher frequency) and phase (for lower frequency) based on the Rihaczek distribution are given by:


(3) $$A_{f_{a}}\left(t\right)=A\left(t\right)e^{j\varphi \left(t\right)}\left[A\left(t\right)e^{-j\varphi \left(t\right)}*sinc\left(f_{a2}-f_{a1}\right)t\right]e^{j\frac{f_{a1}+f_{a2}}{2}t},$$

(4) $$\varphi_{fp}\left(t\right)=\varphi \left(t\right)-\theta \left(f_{p}\right)-2\pi f_{p}t,$$

where f_a1_ and f_a2_ define the high frequency amplitude bandwidth and ϕ(t) and θ(fp) refer to the phase of the low-frequency band in the time and the frequency domains, respectively. In practice, the calculation of the amplitude and phase for each epoch was performed by using the MATLAB code provided in the original publication (Munia and Aviyente, 2019).

The phase-amplitude coupling metric is then calculated using the Modulation Index (MI) (Tort et al., 2009). This method seems to be relatively robust to phase biases (van Driel et al., 2015) compared to other methods such as the mean vector length (Canolty et al., 2006) and quite conservative in low signal-to-noise ratio conditions. MI discretizes the phase angle time series of the phase frequency into *N* phase bins and computes the average power of the modulated frequency for power in each bin *j*. In this work, we set *N* = 18, similar to that used in other studies (i.e., 20° wide bins). Coupling is operationalized in an information-theoretical way as the deviation of the phase-amplitude histogram from the uniform distribution:


(5) $$MI=\frac{D_{KL}\left(P,U\right)}{\log\left(N\right)},$$

where *N* is the number of bins, D_KL_ is the Kullback–Leibler distance between the phase distribution *P* and the uniform distribution *U*:


(6) $$D_{KL}\left(P,U\right)=\log\left(N\right)-H\left(P\right)$$

and *H* is the Shannon Entropy of the phase distribution:


(7) $$H\left(P\right)=-\overset{N}{\underset{j=1}{\sum }}P\left(j\right)\log\left[P\left(j\right)\right].$$

For each epoch, MIs were calculated across the full bandwidth of phase and amplitude bands. For each epoch, MI values across the comodulogram were *z*-scored (zMI) and the maximum zMI was the PAC metric for every epoch. Similar to the band ratio analysis, the average and variance of epoch PACs was taken for every subject. In practice, zMI was applied using a custom-made Python code (which will be made available upon request).

### Deriving supervised spatial filters using common spatial patterns

The Common Spatial Patterns (CSP) algorithm (Koles, 1991; Blankertz et al., 2007; Lotte et al., 2007, 2018) has some similarities with PCA in the sense that it is also an eigendecomposition method. However, instead of finding the filters that maximize uncorrelated signal variance like PCA, its aim is to find filters that maximize the variance for one group of subjects and minimize the variance for the other (i.e., discriminative EEG spatial patterns). Therefore, CSP is a supervised method that calculates spatial filters based on labeled data. Given a set of *t* epoch segments X_t_ ∈ ℝ^dxN^ (where *d* is the number of channels and *N* is the number of datapoints on each epoch), epoch covariances 𝜮_t_ = X_t_X_t_^T^ ∈ ℝ^dxd^, and 𝜮_1_ and 𝜮_2_ as the average epoch covariances for group 1 and group 2 subjects, CSP is calculated by the simultaneous diagonalization of the two average covariance matrices


$$W^{T}\Sigma_{1}W=\Lambda_{1}$$

(8) $$W^{T}\Sigma_{2}W=\Lambda_{2}$$

where W is commonly determined so that $\Lambda_{1}+\Lambda_{2}=I$ (with Λ being a diagonal matrix of eigenvalues). Technically, this is achieved by solving the generalized eigenvalue problem


(9) $$\Sigma_{1}W=\Lambda \Sigma_{2}W.$$

In practice, this was calculated either using MATLAB’s *eig* function or Python *linalg.eigh* function from the *scipy* package. The spatially filtered signal *S* of this set of EEG epoch segments is then given by


(10) $$S=WX_{t}$$

with the leftmost spatial filters of *W* (first column vectors) maximizing the signal variance for group 1 and minimizing the signal variance for group 2, and the rightmost spatial filters (last column vectors) maximizing the signal variance for group 2 and minimizing the signal variance for group 1. For the CSP analyses, each participant’s final score was calculated as the average CSP power of their epochs.

### Linear classifier

A Support Vector Machine with a linear kernel (SVM-L) was the classifier of choice throughout this work. SVMs are useful for data classification as they find the separating hyperplane with the maximal margin between two classes of data. Given a set of data $X_{i}$ with corresponding labels $y_{i}\in \left\{1,-1\right\}$ , SVM solves the unconstrained problem:


(11) $$\underset{W,b}{\min}\frac{1}{2}W^{T}W+c\overset{T}{\underset{i=1}{\sum }}\xi \left(W,b,X_{i},y_{i}\right)$$

where *T* is the number of epochs, $\xi \left(\mathrm{W,b,}X_{i},y_{i}\right)$ is a loss function and c ≥ 0 is a regularization parameter on the training error. The loss function used in this work was a L2-loss:


(12) $$\max{\left(1-y_{i}\left(W^{T}\varphi \left(X_{i}\right)+b\right),0\right)}^{2}$$

where φ is the function that maps the training data into a higher dimensional space in non-linear instances of SVM. In the linear case, however, $\varphi \left(X_{i}\right)=X_{i}$ . Therefore, for any testing instance x , the predictor function for SVM-L is similar to that used in linear discriminant analysis:


(13) $$f\left(x\right)=sign\left(W^{T}x+b\right)$$

To optimize the model training against the class imbalances present on our datasets (especially the story-listening task), the regularization parameter was balanced for each class. Each class weight would take the proportion of class frequencies into account and the new parameter *c*’ is then calculated as $c^{’}=class\_weight\left[i\right]*c.$ In practice, the SVM-L for classification was applied using Python’s *scikit-learn* package implementation (*svm.SVC* function).

Grid search cross-validation was used to optimize not only the regularization parameter but also the number of features to use for the CSP (i.e., filters) as only a subset of the total filters is used (*viz.* the *m* first and last rows of S, i.e., S_p_, *p* ∈ {1…2 *m*}). This cross-validation process was denoted as Leave-One-Subject-Out cross-validation and worked as follows: for each fold, all epochs of a single participant were held for validation while the other epochs were used for training the classifier. In this work, we report the performance of the classifier on the held-out validation data. The label attributed to the child (e.g., dyslexic / non-dyslexic) was based on the majority of classifications for that child’s epochs. For every child, the proportion of story listening epochs classified as “dyslexic” was used to create the receiver operating characteristic (ROC) curves. The feature estimation process was also cross-validated between 1) variance, 2) log variance and 3) proportional variances as in (Ang et al., 2008):


(14) $$X^{*}{\;}_{p}=\left(\frac{\mathrm{var}\left(S_{p}\right)}{\sum_{i=1}^{2m}\mathrm{var}\left(S_{i}\right)}\right).$$

Beta band responses were also included in the classifier analyses, following Keshavarzi et al. (2024). For both datasets, the variance features worked best. For all classifiers, the other hyperparameters were kept at *m* = 2, *c* = 100 as determined by grid-search cross-validation. During this feature engineering process we also found that normalizing variables by a power of 10 helped model convergence in some cases. This was the case for the story listening task, where this constant was set to 10e4 after grid-search cross-validation.

## Results

### Do children with dyslexia show different theta/delta oscillatory relations during natural speech processing?

Our first research question was whether the relationships between low-frequency oscillations during natural speech processing are different in children with developmental dyslexia (Figure 1A). We focused on delta (1-4 Hz) and theta (4.5-8 Hz) power dynamics, and investigated differences between the Australian typically-developing children, children with dyslexia and children with DLD who participated in the story listening task (see Table 1). To focus the analysis, we placed emphasis on the uncorrelated brain regions that provided the highest differential response to the listening task. To identify these regions, we made use of Principal Component Analysis (PCA) and computed band power from each PC individually. We retained only PC filter vectors with the highest eigenvalues, setting the threshold at a total of 70% variance explained (see Methods for further details). Applied to the current dataset, this translated to just three PCs (see Figure 1B). Their associated channel weights showed stereotypic patterns consistent with the audio-visual nature of the experimental task: PC1 (total variance explained: 50%) showed an enrichment of channel weights in the central region of the scalp, where evoked auditory potentials are usually observed. PC2 (total variance explained: 15%) showed a pattern of bilateral temporal electrodes covering both auditory cortices. Finally, PC3 (total variance explained: 13%) lay mostly on occipital channels.

Theta/delta power ratios were calculated to test for the relative magnitude of theta oscillations compared to delta oscillations across groups. As many ratio distributions did not show a normal distribution (based on Shapiro–Wilk tests), non-parametric Kruskal-Wallis ANOVAs and post-hoc Wilcoxon tests were used to assess group differences. Two metrics were evaluated for each child for PC power ratios across the experimental session: the mean ratio value, to check for a general difference in the band power relationship trend; and the variance of this ratio, to test for differences in consistency for this relationship. The results are shown in Figure 1C. For PC1, group differences were found on the mean theta/delta ratio across groups (*H* = 10.96, *p* = 0.0042). Post-hoc Wilcoxon tests showed a higher mean theta/delta ratio for dyslexic children when compared to typically-developing children (*Z* = −2.87, *p* = 0.004) and DLD groups (*Z* = 2.77, *p* = 0.0056). Importantly, these differences did not arise because of a general difference in delta (*H* = 2.68, *p* = 0.26) or theta power (*H* = 1.39, *p* = 0.5). Group differences were also found regarding the consistency across epochs for theta/delta ratio variance (*H* = 11.49, *p* = 0.0032). Overall, ratio variances were higher for dyslexic children compared to typically-developing children (*Z* = −3.26, *p* = 0.0011). Differences in ratio variance between dyslexics and the small group of children with DLD were not significantly different following Bonferroni correction (*Z* = 2.24, *p* = 0.0252). The children with DLD did not differ from typically-developing children regarding the power ratio consistency and mean value for PC1 (*p* > 0.05 for both), and no group differences were found for the average value or consistency of any other PC filters (*p*’s > 0.05). Taken together, these results suggest an atypically high and less consistent power dynamic between delta and theta oscillations for children with dyslexia. These differences are present in centrally-located regions of the scalp.

To explore whether individual differences in both reading and phonology were related to these oscillatory dynamics, Spearman correlations between the mean theta/delta ratio for each child for PC1 and both their phonological awareness and word reading ability were computed (Figure 1D). For dyslexic children only, a significant relationship was found for phonological awareness (*r*_s_ = −0.55, *p* = 0.027) but not for reading (*r*_s_ = 0.03, *p* = 0.916). This suggests a dyslexia-specific pattern of interplay between delta and theta power during natural speech processing that is progressively less atypical the better the child’s phonological awareness. To further control for PC1 sensitivity to developmental and reading level effects, typically-developing children were divided into chronological age controls (matched in age to the dyslexic children, but with better reading skill) and reading level controls (similar reading level to the dyslexic children, but over 2 years younger, see Table 1). The CA and RL control groups were compared to see whether they showed significantly different mean and variance regarding their theta/delta ratio. No significant differences were found across these subgroups (Figure S3). Correlations between PC1 theta/delta ratio and age; and PC1 theta/delta ratio and general IQ were also calculated. Once again, no significant relationships were found for any group (*p’s* > 0.05 for all correlations).

### Do dyslexia-specific theta/delta power ratio differences for speech processing transfer to the rhythmic syllable repetition task?

**Figure 2:**
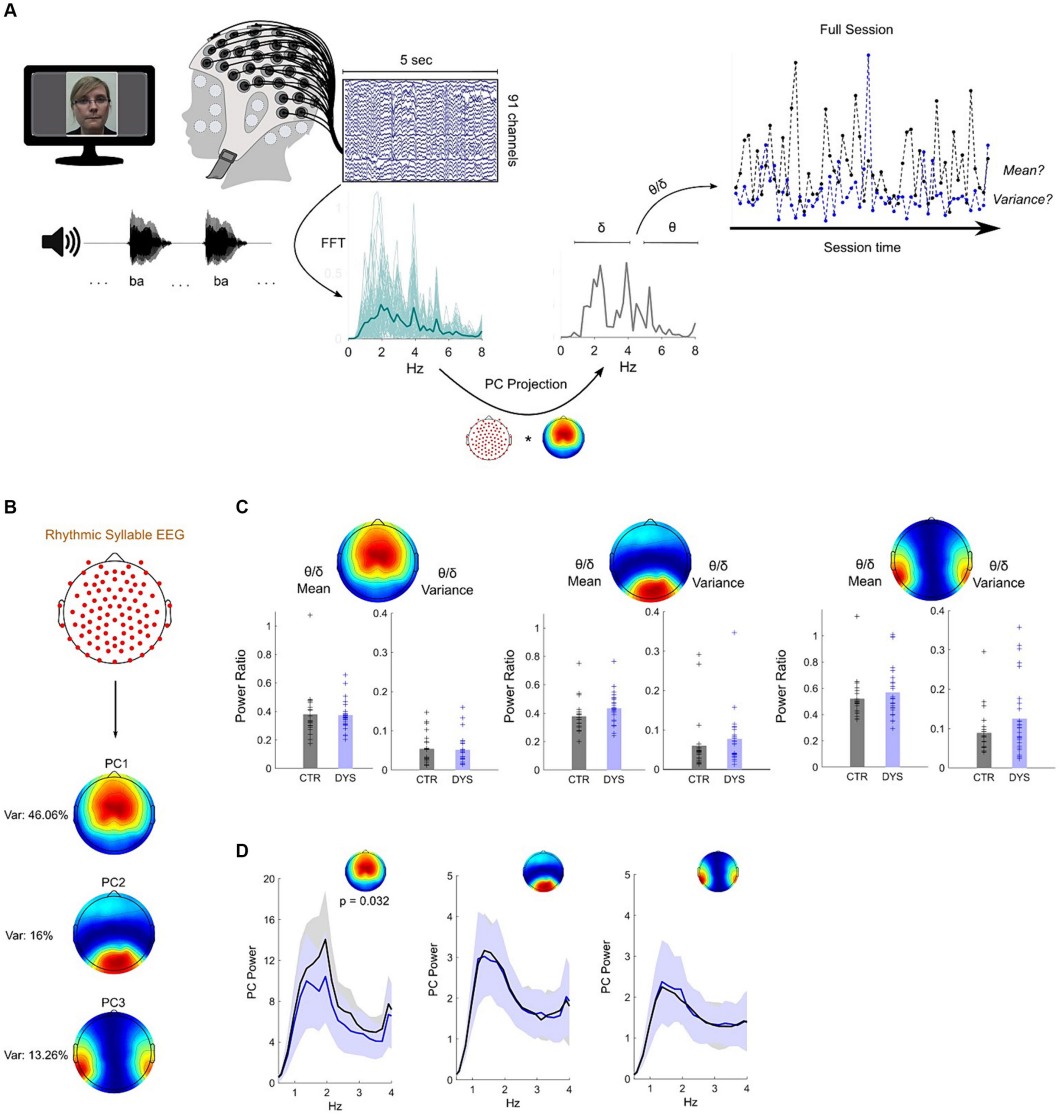
Dyslexia-specific theta/delta power ratio differences for speech processing do not transfer to rhythmic syllable repetition. **(A)** Paradigm and pseudo-online analysis pipeline (see Methods). EEG projection to PC1 was used for illustration. Sessions from a dyslexic (blue) and a typically-developing child (black) are shown. **(B)** Map of the three most important principal components – closely matching those of the story listening task (see Figure 1B). **(C)** No group differences were observed across all three PCs for theta/delta ratio mean or variance (CTR – typically-developing children, DYS – dyslexic children). **(D)** Typically-developing children show higher delta band power on PC1 only, which was maximal at the syllable presentation rate (2 Hz).

Our second research question was whether these power modulation differences in dyslexia were specific to connected speech. This was achieved by analysing a second EEG dataset recorded while British children with dyslexia and TD controls performed an audio-visual speech task without semantic or syntactic content (Figure 2A). The task involved repetition of the syllable “ba” by a ‘talking head’ (see Methods). PCA was again used to derive spatial filters for calculating theta/delta ratios in distinct brain regions. As with the story listening task, three PCs were sufficient to account for >70% of the variance (Figure 2B). Crucially, the spatial filters derived for the rhythmic repetition task shared remarkable similarities in terms of the spatial organisation and relative percentage of variance explained with the story listening task (compare Figure 2B to Figure 1B). The syllable repetition task showed a dominant central PC1 (total variance explained: 46%) followed by occipital (total variance explained: 16%) and bilateral temporal (total variance explained: 13%) PCs. The main difference between the syllable repetition and story listening task components was that the spatial configuration of PC2 in one task mirrored that of PC3 for the other task and *vice-versa*.

To investigate whether the rhythmic syllable repetition task would also show a group theta/delta ratio difference, group means and variances were compared. No group differences were found either for theta/delta mean or variance on any of the three dominant PCs (*p*’s > 0.05 for all comparisons) suggesting the theta/delta ratio effect is specific to connected speech (Figure 2C). However, in a further analysis for PC1, we found a significant group difference in delta band power (*Z* = 2.14, *p* = 0.032). Specifically, inspection of the EEG power spectra at 1-4 Hz (Figure 2D, left panel) showed that the peak difference occured at 2 Hz – the “ba” repetition rate – with a stronger response for typically-developing than dyslexic children. These data indicate a difference in neural steady-state responses to the rhythmic syllable targets. No other PCs showed this effect (Figure 2D, middle/right panels). Overall, these results suggest that differences regarding the interplay of delta and theta power dynamics in dyslexia do not transfer to a speech listening paradigm that lacks semantic / syntactic content or phrasal structure. Nevertheless, as found in previous neural investigations, the EEG delta band response is different in children with dyslexia.

### Does delta-theta cross-frequency coupling (CFC) during natural speech listening vary across groups?

**Figure 3:**
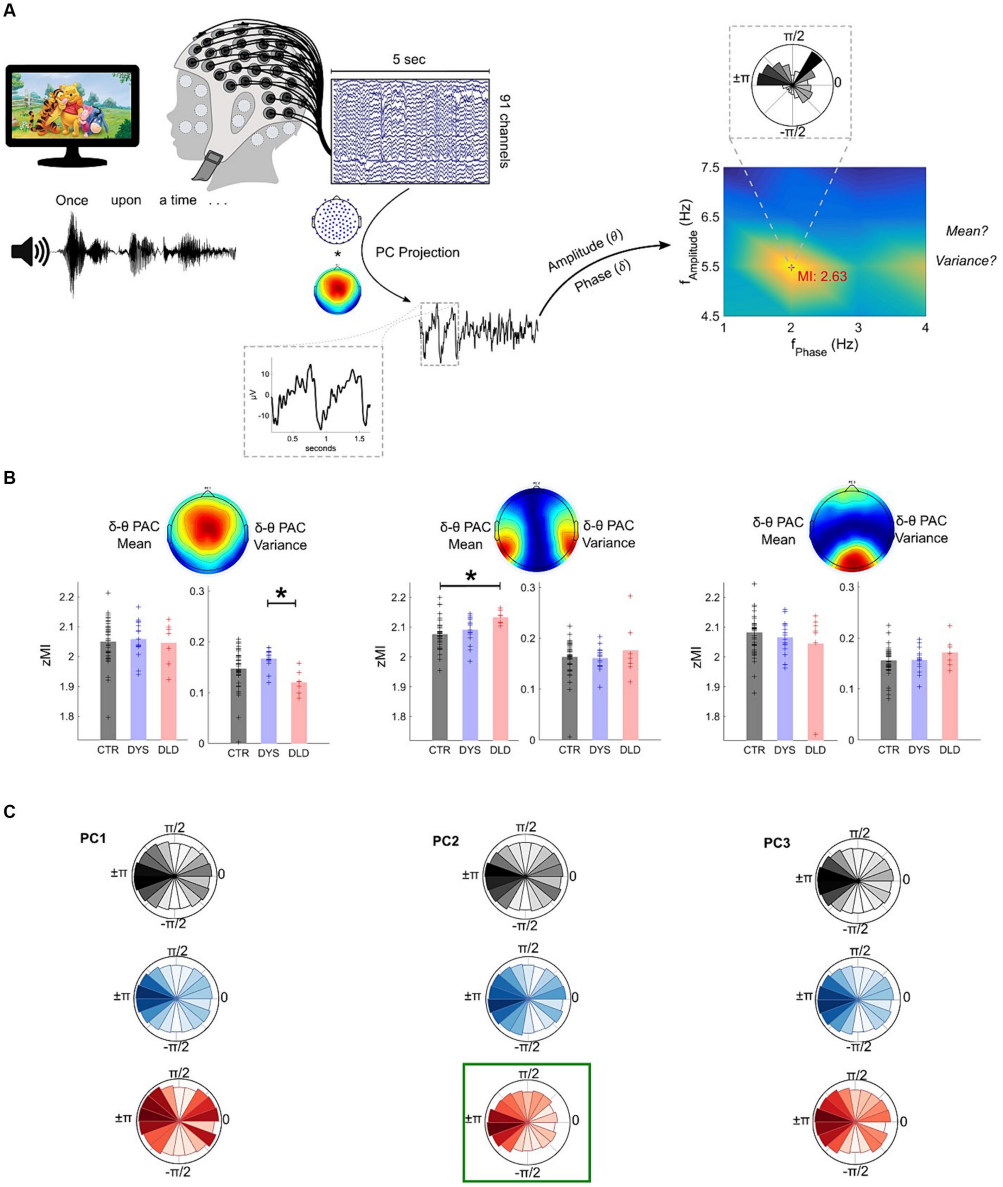
DLD children exhibit atypical delta-theta Phase-Amplitude Coupling (PAC) during speech listening (story listening task). **(A)** Analysis pipeline example using a single epoch. The comodulogram with the z-scored Modulation Index (zMI) values and phase-amplitude polar plots of maximum cross-frequency coupling are shown. **(B)** Phase-Amplitude Coupling (PAC) results. DLD children show significantly lower variance than dyslexic children for PC1 and significantly higher mean than typically-developing children for PC2. * *p* < 0.05 (Bonferroni corrected). **(C)** Polar plots showing the concentration of theta amplitudes across delta phase bins. A relatively higher concentration of high theta amplitudes on PC2 at ±π can be observed for DLD children (highlighted in green) when compared to the other groups.

A related question was whether *cross-frequency coupling* of low-frequency oscillations – a neural mechanism crucial for sensory selection in speech processing – would potentially differ across groups. Accordingly, phase-amplitude coupling (PAC) differences involving the bands of interest were investigated using the story listening data. Principal Component Analysis was again used to spatially filter whole-brain data and delta-theta PAC was calculated for all children. Phase and amplitude of lower and higher frequencies, respectively, were computed using a time-frequency method that does not utilize bandpass filters, and a z-scored modulation index (*zMI*) was used to estimate neural PAC (see Methods). For each participant, the average value and consistency (variance) of the coupling metric across the experimental session was calculated. The analysis pipeline is depicted in Figure 3A and mean / variance group comparisons of delta-theta PAC are shown in Figure 3B. While maximum coupling for most children occurred in the lower half of the delta band (1-2 Hz) phase, the amplitude frequency for maximum PAC was highly variable across the theta band range (Supplementary Figure S4). In Figure 3C (all panels) it is clear that this coupling occurs at a preferred delta phase of around ± π across the 3 dominant PCs for all groups.

For PC1 (which had the largest channel weights in centrally-located electrodes), group differences were found for *zMI* variance (*H* = 12.1, *p* = 0.0024). Wilcoxon tests comparing TD children and dyslexic children (*Z* = -2.1, *p* = 0.036) or TD children and children with DLD (*Z* = 2.3, *p* = 0.022) were not significant following a Bonferroni correction. However, the two clinical groups showed significantly different *zMI* variances (*Z* = 3.3, *p* = 9.4e-4), with children with dyslexia showing higher variance in maximum coupling compared to children with DLD across the experimental session (Figure 3B, left panel). Phase-amplitude plots (Figure 3C, left panel) indicate a relatively higher concentration of large amplitudes around the 0:π/4 phase of the delta band for the small group of children with DLD. No significant differences were found in average *zMI* for PC1 across groups (*p* > 0.05).

For PC2 (largest channel weights for electrodes covering temporal areas), differences were found across groups for mean *zMI* (*H* = 11.53, *p* = 0.0031). The children with DLD showed higher delta-theta coupling (*Z* = −3.2, *p* = 0.0016) compared to typically-developing children (Figure 3B, middle panel). Phase-amplitude plots (Figure 3C, middle panel) showed that this significantly greater phase-amplitude coupling for children with DLD stemmed from a higher concentration of larger theta amplitudes for the delta ±π phase bin, where larger amplitudes are also seen for the other groups. *zMI* mean comparisons between TD children and children with dyslexia (*Z* = −1.5, *p* = 0.125) and between children with dyslexia versus DLD (*Z* = −2.2, *p* = 0.025) were not significant after a Bonferroni correction. Delta-theta variance of *zMI* for PC2 was similar across all groups (*p*’s > 0.05). No differences regarding maximum coupling strength across groups were found for PC3, the spatial filter with the largest weights on electrodes covering the occipital cortex (*p*’s > 0.05).

Finally, to test the sensitivity of PC1 and PC2 delta-theta PAC to developmental or reading level effects, we again divided the typically-developing Australian children into subgroups of chronological age and reading level matched controls. No significant differences were found between these subgroups for delta-theta PAC (mean or variance) on either PC1 or PC2 (Figure S5).

Taken together, these results show that PAC metrics can distinguish children with DLD from groups of both TD and dyslexic children. These differences in cross-frequency coupling were observed in principal components covering auditory / central areas. As was the case also regarding theta/delta power ratios, no group differences were observed in principal components covering primary visual areas.

### Can distinct patterns of oscillatory dynamics enable discrimination between dyslexic, DLD and typically-developing children during natural speech listening?

**Figure 4:**
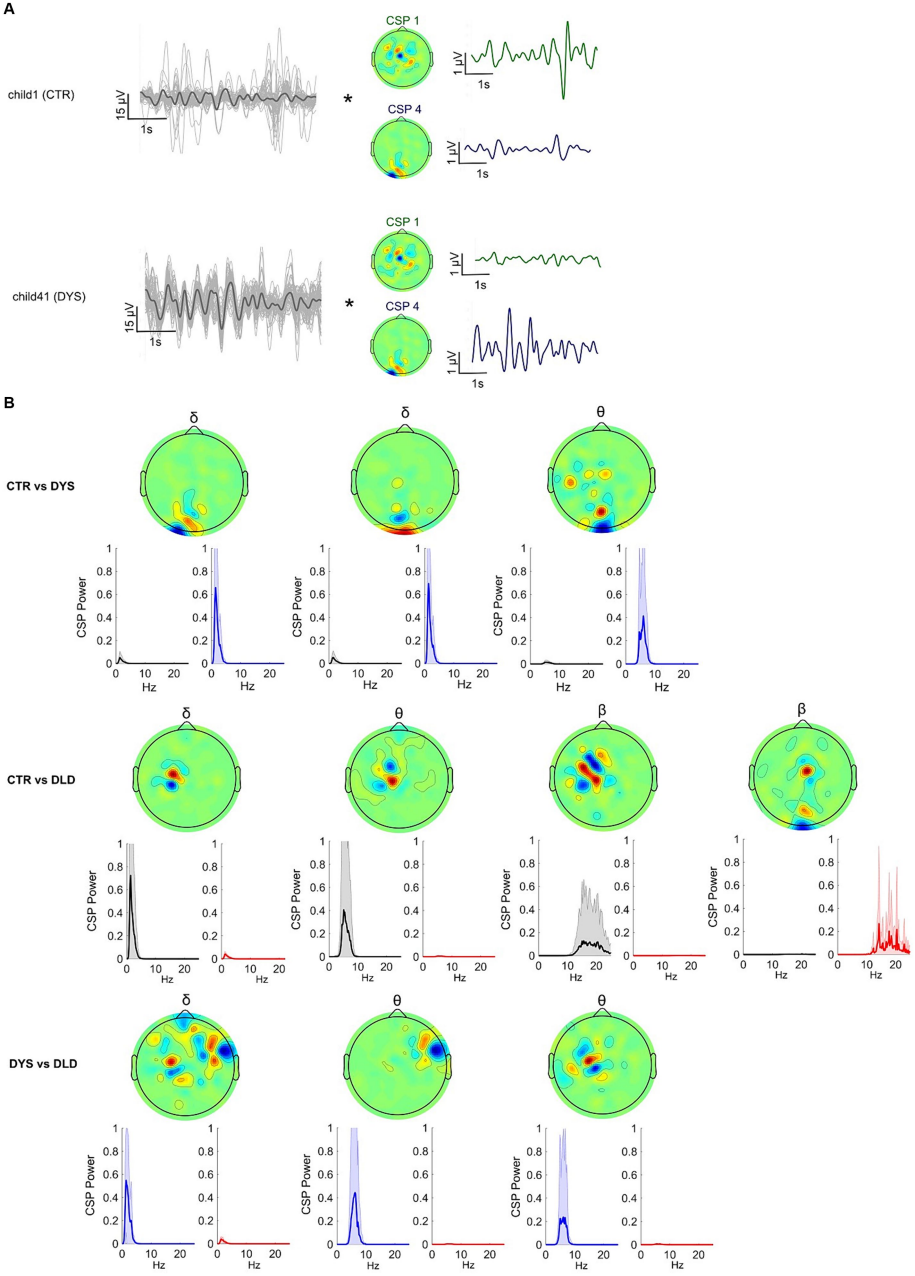
Common Spatial Patterns (CSP) enable discrimination between dyslexic, DLD and typically-developing children during speech listening (story listening task). **(A)** Spatial filtering applied to the original EEG epochs (each channel depicted in grey, average in black). Spatial filters allow discrimination of EEG signals from experimental groups in the story listening task by selectively maximizing the signal variance for one group while minimizing for the other. **(B)** CSP filters for delta, theta and beta showing average power for each group for each filter. These CSPs showed significant differences at the group level (*p* < 0.05, Bonferroni corrected). Upper panel shows filters yielding significant group differences for the typically-developing children vs. dyslexic, middle panel for the typically-developing vs. DLD children and lower panel for the dyslexic vs. DLD children.

Our third research question was whether distinct patterns of oscillatory dynamics during natural speech listening would be robust enough to build a classifier for dyslexia. The theta-delta ratio and PAC results suggest that there are distinct atypical patterns of low-frequency oscillatory dynamics during natural speech listening for Australian children assigned to the dyslexia and DLD groups. However, potential group differences in spatial patterns regarding each individual oscillatory frequency have not yet been addressed. We therefore used the story listening data to seek biomarkers that could show group differences across delta and theta oscillations, and also included beta oscillations (beta oscillations were included following Keshavarzi et al., 2024). Previous Brain-Computer Interface studies have used Common Spatial Patterns (CSP, see Methods) to find optimal EEG patterns successfully. Applied to the present study, this linear method finds supervised filters (using labeled data) that simultaneously maximize the EEG signal variance for one group of children while minimizing the variance for the other, and *vice-versa* (Figure 4A). To uncover spatial filters that would discriminate between our three groups, 3 sets of CSP filters were calculated for delta, theta and beta oscillations, respectively, (translating to a total of 9 sets). Specifically, we compared (1) typically-developing versus dyslexic children, (2) typically-developing versus DLD children, and (3) dyslexic versus DLD children for each oscillatory band. Each CSP calculation yielded the same number of filters as the number of recorded channels. As in previous BCI research, only a subset of these filters was analyzed. For each group comparison, the 2 spatial filters maximizing the variance for each group (i.e., for *m* = 2; total number of filters = 4) were computed and participant differences were assessed. Twelve comparisons were made (4 spatial filters x 3 brain rhythms) for each group combination and only the filters showing significant differences in non-parametric ANOVAs that survived Bonferroni correction were considered (threshold *p*-value = 0.05/12 = 0.004).

Figure 4B depicts the results. When comparing typically-developing Australian children with children with dyslexia (Figure 4B, upper row), filters relying mostly on *occipital* channels were the most discriminative. These filters maximized the EEG power for children with dyslexia on delta and theta rhythms. When comparing typically-developing children with children with DLD (Figure 4B, middle row), central / left lateralized CSP filters maximized the EEG power for typically-developing children (minimizing for DLD children) across all 3 brain rhythms. A spatial filter focusing on occipital channels minimized the variance for typically-developing children while maximizing variance for children with DLD in the *beta band* range. Overall, strongly occipital EEG filters minimized the EEG power of typically-developing children while maximizing power for both DLD and dyslexic groups (see top and middle rows, Figure 4B). Finally, when comparing dyslexic with DLD children (Figure 4B, bottom row), the significant spatial filters were on delta and theta rhythms. These CSPs maximized the EEG power for children with dyslexia and had strong weights on the right temporal and left central channels.

### Can a linear classifier trained with features based on common spatial patterns identify children with dyslexia?

**Figure 5:**
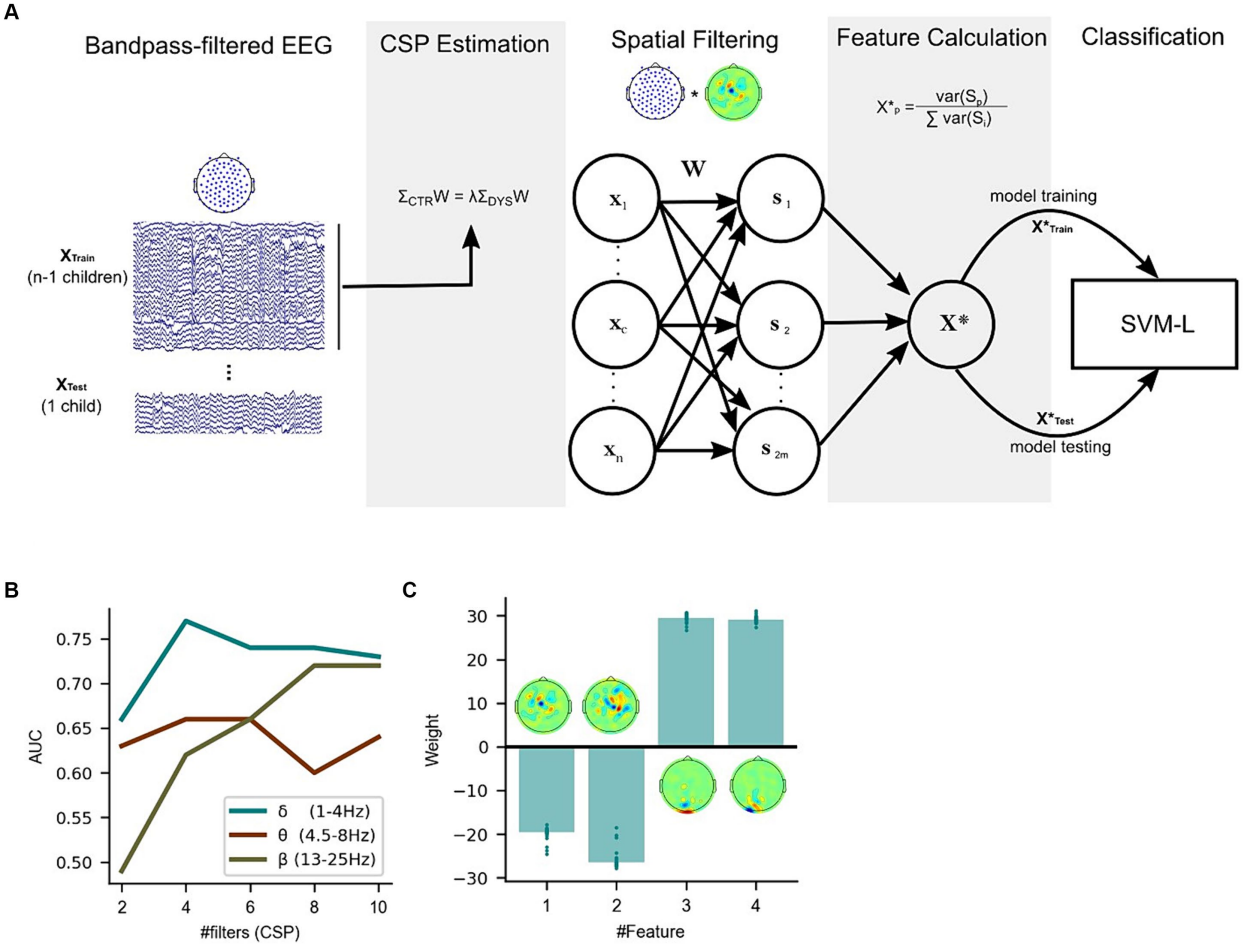
Classification of dyslexia using a linear classifier trained with features based on Common Spatial Patterns on the story listening task dataset. **(A)** Leave-one-subject-out cross-validation pipeline for the Linear SVM algorithm (total number of features = 2 *m*). **(B)** Performance (based on the area under the curve – AUC) difference between linear classifiers using delta, theta and beta-CSP filters across a different number of features (#filters). **(C)** Delta-CSP feature weights of the linear classifier are stable across cross-validation folds.

Despite the significance of between-group differences for CSP-based biomarkers, it is not yet clear whether features based on these CSPs have enough robustness to be used in a classifier – especially for the 5-s window epochs employed here. To test this robustness, we cross-validated linear Support Vector Machines – with delta, theta and beta filtered EEG CSP feature inputs, respectively – using Leave-One-Subject-Out cross-validation. This method allows repeated assessment of the model’s performance without leaking participant-specific information to the training set (see Figure 5A and Methods). SVMs have been frequently used as classifiers in the EEG literature and have shown performances ranging from 0.6–0.95 AUC in EEG classification problems using longer inputs (Gallego-Molina et al., 2022). Here each classifier was trained with a different number of CSP filters to detect the optimal number of features. Importantly, for each cross-validation fold, CSPs were calculated only on the training set to avoid feature information leakage from the test set and, consequently, overfitting. To avoid “double dipping,” no information from previous group analyses was used to influence the choice of specific spatial filters (see Methods).

The receiver operating characteristic (ROC) curves for the classifiers are presented in Figure 5B. Simple linear SVMs using 4 feature variables (i.e., spatial filters) with the delta-CSP classifier reached an area under the curve (AUC) of 0.77. Since this delta-CSP classifier showed the best performance (compared to all the theta- and beta-CSP classifiers, see Figure 5B), we further analyzed the spatial location of its most important CSP weights. We also analyzed the relationship between these CSPs and EEG activity, and the variance of the classifier’s weights for the CSP features across all 56 folds (defined as the number of participants in Leave-One-Subject-Out cross-validation). The results showed that the two negative-weighted CSP features were those that maximized the signal variance for typically-developing children and minimized the variance for children with dyslexia (Figure 5C). These spatial filters were more strongly weighted on central / right lateralized channels and showed relatively higher correlations with channel activity in left/frontal and right/central areas of the scalp for control children when compared to children with dyslexia (Supplementary Figures S6A,B). On the other hand, the largest positive weights were the two spatial filters that maximized the signal variance for children with dyslexia and minimized the variance for typically-developing children. These filters were mainly focused on occipital channels, and the absolute values of their weights were similar to those of other spatial filters. These occipital CSP filters showed stronger correlations with electric potentials in larger areas of the scalp for children with dyslexia (versus typically-developing children), extending to parietal and central electrodes (Supplementary Figure S6C,D). Feature weights remained stable across the cross-validation process (Figure 5C), despite CSP filters being slightly different for every fold (given the changes in the training set). CSP channel weight estimations across folds were also highly consistent (Supplementary Figure S7).

### Will delta-CSP features for dyslexia classification transfer across from story listening to the rhythmic syllable repetition task?

**Figure 6:**
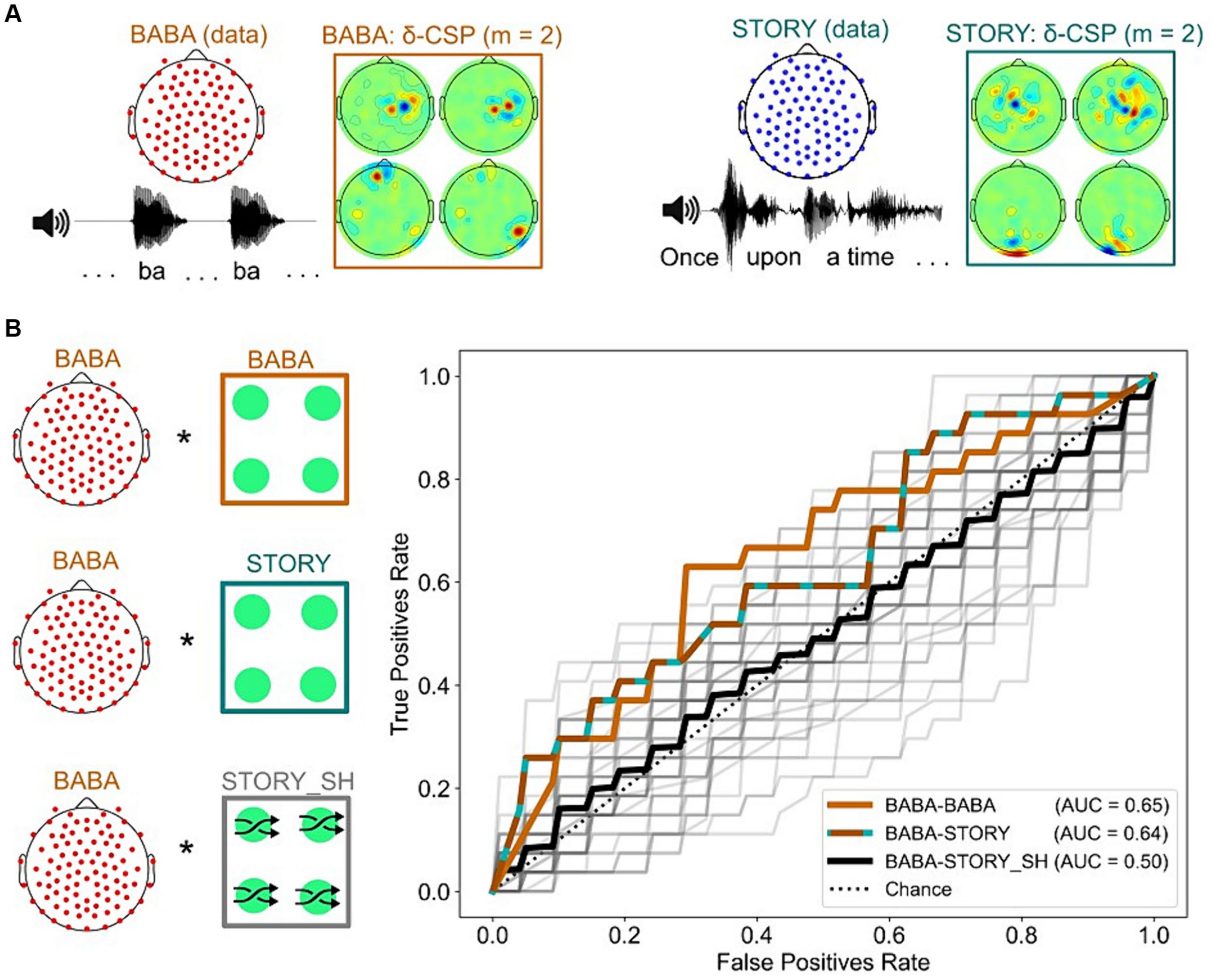
Analyses revealing successful transfer-learning of delta-CSP features for dyslexia classification across story listening and rhythmic syllable repetition tasks. **(A)** Delta-CSP filters for the rhythmic syllable task (orange) show higher weights on right temporal channels when compared to the delta-CSP filters of the story-listening task (teal) that show higher weights on occipital / central channels. **(B)** Similar AUCs for linear SVMs classifying dyslexics and typically-developing children on the rhythmic syllable repetition task with its original cross-validated CSPs (BABA-BABA) and with CSPs derived from the story listening task (BABA-STORY). SVMs using spatially shuffled versions of the story CSPs (BABA-STORY_SH) resulted in chance-level performance (black).

Our final research question was whether a reliable classifier could be based on the same neural features for natural connected speech and simplified rhythmic speech Given the apparently central role of delta-band responding for dyslexia, we investigated whether these same 4 delta-band spatial filters that identified dyslexia in story listening EEG might contain useful information for classifying dyslexia in the rhythmic syllable repetition EEG. We thus trained a linear SVM classifier to predict dyslexia on the rhythmic syllable repetition dataset, using 4 delta-CSP filters trained using the story listening dataset (Figure 6A). To obtain baseline performance for a classifier trained on the most discriminative CSP filters computed for the syllable repetition task, a pipeline similar to the story listening task (see Figure 5A) was applied to this dataset.

Figure 6B shows the results. SVM performance regarding classification of dyslexic children on the rhythmic syllable repetition data with the original CSP filters versus the story-listening CSPs was very similar (0.64 and 0.65 AUC, respectively). However, this generalization may occur simply because temporal filtering (i.e., the 1-4 Hz bandpass for delta) is the sole provider of a whole-brain metric for classification, irrespective of spatial filter weights on specific channels. To test this possibility, we trained 50 linear SVMs, each one using a different random permutation shuffle of the story-listening CSP weights (BABA-STORY_SH condition, see also Figure S8). The average AUC performance of these classifiers was at chance level (black ROC curve, AUC = 0.5), and the model using the original story-listening CSPs was better than 96% of models using its shuffled versions at predicting dyslexia status from the rhythmic syllable repetition task (Figure 6B, grey ROC curves). This result shows that the specific configuration of story-listening EEG spatial filters is encoding important information for classifying dyslexia using the rhythmic syllable repetition EEG. Accordingly, speech rhythm processing may be the common factor being identified by our classifiers regarding dyslexia and potential biomarkers.

## Discussion

Here we show that atypical automatic low-frequency neural oscillatory responses to natural speech can uniquely identify children with developmental dyslexia. We further show that low-frequency oscillatory activity during speech listening can reliably classify which children have dyslexia (AUC 77%). Taken together, these results show that dyslexia classifiers can be based on EEG-CSP features. Furthermore, in line with prior neural child studies, delta-band features were found to be most useful to identify developmental dyslexia. The data from the small group of control children with a different linguistic disorder (DLD, *N* = 7) appear to indicate that low-frequency neural oscillatory responses to connected speech can also distinguish between the two most common developmental disorders of language processing, dyslexia and DLD. However, replication of the effects reported here with larger groups of DLD children is required.

These data suggest that mechanistic relationships between low-frequency (i.e., delta and theta) oscillatory bands are fundamental to understanding the aetiology of dyslexia, as predicted by TS theory (Goswami, 2011, 2015, 2022b). The data also show the importance of studying *children* when trying to understand causal factors in developmental disorders of learning. Adult studies have assumed that faster-rate (phonemic, >30 Hz) speech envelope modulations should be impaired in individuals with dyslexia in alphabetic orthographies, as grapheme-phoneme conversion is fundamental to reading proficiency (Lehongre et al., 2011). However, developmental studies show (a) that infants extract phonetic information from natural speech using low-frequency oscillations (di Liberto et al., 2018) and (b) that *phonemic* information is learned via *reading experience*, with fast-rate oscillations showing atypical patterns in children only *after* the onset of reading (Vanvooren et al., 2017; Mandke et al., 2022). Indeed, in their MEG study of natural speech listening, Mandke et al. reported that minimal gamma-band synchronization was present at the beginning of the dyslexic reading trajectory. Further, phoneme-level processing of speech is not related only to faster neural oscillations, as slower oscillations <8 Hz also yield phoneme-level information [e.g., about phonetic features, see Di Liberto et al., (2015, 2018)]. Using EEG, Di Liberto et al. (2018) showed that atypical speech entrainment in the right hemisphere for low-frequencies was associated with phoneme-level processing in dyslexia.

Low-frequency oscillations usually signal communication involving large populations of neurons in relatively large brain areas, while higher frequency oscillations are nested in these rhythms and act more locally (Buzsáki, 2019). The potential EEG markers found here for dyslexia and DLD were indeed spread across large areas of the scalp. The direct mechanistic consequence of such nesting is that atypical oscillatory patterns at lower frequencies (observed here in children with both dyslexia and DLD) may have downstream effects regarding the magnitude of oscillatory responses in high-frequency brain rhythms. Therefore, our data are compatible with previous adult dyslexia studies indexing atypical fast-rate oscillatory power for non-speech steady-state stimuli (Lehongre et al., 2011). The loci found here for atypical low-frequency oscillatory responses are also in line with clinical observations of dispersed structural brain abnormalities in dyslexic participants (Eckert, 2004) as well as molecular differences in genes that regulate the development of the entire language network in both DLD and dyslexia (Fisher and DeFries, 2002; Bishop, 2006; Newbury et al., 2010).

A key finding in our study is that the *relative* oscillatory power of theta/delta responses is atypical in children with dyslexia during a story listening task. Such differences were seen for an electrode ensemble strongly weighted around the centre of the scalp. These loci are consistent with the hypothesis that children with dyslexia are accessing a mental lexicon that contains atypical auditory representations with less accurate representation of delta-band speech envelope information (Power et al., 2016; Destoky et al., 2020, 2022; Mandke et al., 2022; Keshavarzi et al., 2022a). Delta-band envelope information is crucial for perceiving prosodic structure (Leong and Goswami, 2015; Ghitza, 2016; Meyer et al., 2017). Cognitive processing of prosodic information is impaired in children with dyslexia when compared to both age-matched and reading-level matched (hence younger) controls (Goswami et al., 2010, 2013). In line with these prior behavioral findings, here we find this oscillatory marker to be associated with performance in phonological rather than reading measures. These atypical patterns of neural processing are likely to influence the mental representation of prosody in language from infancy and throughout development (Attaheri et al., 2022).

When given a rhythmic syllable repetition task, the children with dyslexia no longer showed a difference compared to typically-developing children regarding the theta/delta oscillation power ratio. In prior work, this rhythmic speech task has revealed atypical *preferred phase* in the delta band in the dyslexic child brain (Power et al., 2013; Keshavarzi et al., 2022b). Here, children with dyslexia showed significantly lower oscillatory delta band power in this task, with maximal differences at the syllable presentation rate (2 Hz). Given the audio-visual nature of the task, this finding suggests that steady-state responses from the dyslexic brain are weaker even when the task allows supra-additive responses due to congruent information across visual and auditory modalities (Schroeder et al., 2008; Arnal et al., 2011; Pattamadilok and Sato, 2022).

We further observed atypical neural cross-frequency PAC for delta-theta in children with DLD but not in children with dyslexia (story listening task). This effect was observable on electrode ensembles covering bilateral temporal regions, which may suggest that the sensory representation / binding of sound features is affected in children with DLD. This explanation is in line with studies showing atypical auditory sensory processing in DLD regarding many non-speech acoustic parameters, including ART as well as frequency and duration (e.g., Corriveau et al., 2007; Beattie and Manis, 2012). A recent MEG study found that children with DLD showed atypical cortical tracking of single words in the theta band (Nora et al., 2024). An unusually strong neural dependence of delta-theta coupling to process speech could potentially explain these difficulties for the DLD children. However, given the small sample size, these observations must remain tentative.

We also found unexpected group differences in occipital regions. Our CSP filtering approach revealed that filters with larger weights on channels covering occipital areas consistently showed group differences between TD children and both children with dyslexia (delta and theta bands) and children with DLD (beta band), even though the story listening task did not provide visual speech information. The developmental disorder groups showed notably high oscillatory activity in occipital regions. While occipital areas are not typically associated with acoustic/phonological deficits, these findings are compatible with a previous developmental literature suggesting compensatory visual mechanisms accompanying auditory processing deficits in children with dyslexia (Arns et al., 2007). Prior EEG speech studies in our own lab have also shown occipital foci (Power et al., 2016). These occipital loci may also pertain to the automatic integration of orthography and phonology as literacy is taught, potentially representing an oscillatory footprint for the visual word-form area (VWFA). The VWFA is activated during receptive language processing by adults (Hickok and Poeppel, 2007), and functional changes have been found in the VWFA for children with dyslexia (Van der Mark et al., 2009; Kast et al., 2010). Meanwhile, children with DLD also showed weaker oscillatory activity across both delta and theta rhythms when CSPs with strong weights for left-lateralized temporal and central channels were used. Left-lateralised effects are typical in the fMRI neuroimaging DLD literature (Asaridou and Watkins, 2022, for recent review). The beta band differences were not anticipated, nevertheless the magnitude of beta oscillatory responses has been associated not only with comprehension but also with predictive coding of speech (Gisladottir et al., 2018). Taken together, these findings may suggest that speech processing and speech prediction are atypical in children with DLD, a clearly different phenomenological manifestation from that observed in children with dyslexia.

Large non-linear EEG-based classifiers have previously been engineered for dyslexia using long time windows of AM noise, with degrees of success reaching an AUC ~ 0.8 (Ortiz et al., 2020; Gallego-Molina et al., 2022). By contrast, we tested classification performances for both dyslexic and TD children using a linear classifier and short epochs (~5 s) of naturalistic speech listening data. We found features from a minimal number of EEG spatial patterns for delta oscillatory responses, which showed high-magnitude differences between dyslexic and neurotypical children. We then trained dyslexia classifiers for the less naturalistic rhythmic syllable repetition task using CSPs from the story task, enabling transfer-learning across datasets. Crucially, these classifiers showed performances comparable to classifiers trained with their original CSP features. This appears to suggest that we are picking up oscillatory patterns related to general speech rhythm processing. Interestingly, half of the CSPs from the syllable repetition task were specifically located in right-lateralized temporal areas, an area typically active during prosodic tasks (Sammler et al., 2015). This indicates differences in the symmetry of spatially filtered delta oscillations between dyslexic and typically-developing children, matching adult data (Hämäläinen et al., 2012; Lehongre et al., 2013).

In conclusion, we have identified relationships between low-frequency EEG oscillations related to different neural speech processing mechanisms that are selectively atypical in dyslexia. Further, we find that the magnitude of delta oscillations in a story listening task shows a consistently different pattern between dyslexic and typically-developing children, potentially enabling the development of a generalizable classifier for developmental dyslexia. Our cross-dataset approach provides evidence that these oscillations are likely related to speech rhythm processing, a core tenet of TS theory. We also demonstrate transfer-learning of EEG features for identification of children with dyslexia across different receptive speech tasks and different samples of children. Taken together, our data provide robust evidence of the utility of employing a temporal sampling framework to explain developmental disorders of language learning.

## Data availability statement

The raw data supporting the conclusions of this article will be made available by the authors, without undue reservation.

## Ethics statement

The studies involving humans were approved by the Cambridge Psychology Research Ethics Committee. The studies were conducted in accordance with the local legislation and institutional requirements. Written informed consent for participation in this study was provided by the participants’ legal guardians/next of kin.

## Author contributions

JA: Conceptualization, Methodology, Formal analysis, Visualization, Software, Data Curation, Writing – original draft. BS: Conceptualization, Supervision, Writing – review & editing. VP: Investigation, Writing – review & editing. KM: Investigation, Writing – review & editing. MK: Investigation, Writing – review & editing. AM: Investigation, Writing – review & editing. FG: Investigation, Writing – review & editing. AW: Investigation, Writing – review & editing. GD: Investigation, Writing – review & editing. DB: Funding acquisition, Resources, Writing – review & editing. UG: Conceptualization, Funding acquisition, Supervision, Resources, Writing – original draft.
