# Supplementary figures and images for: Atypical low-frequency cortical encoding of speech identifies children with developmental dyslexia
Source: Front Hum Neurosci. 2024 Jun 7;18:1403677. doi: 10.3389/fnhum.2024.1403677 (PMC11190370; doi:10.3389/fnhum.2024.1403677)

**a**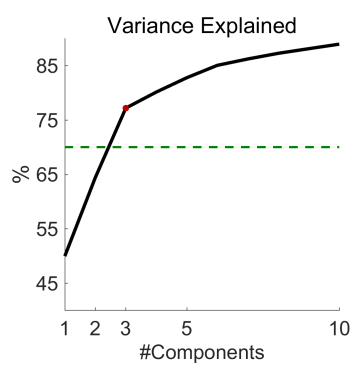**b**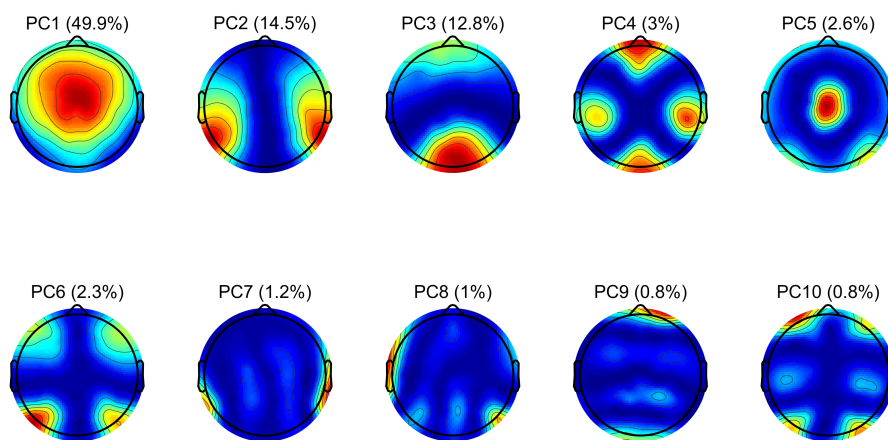

Supplement: Supplementary file 1 [file Data_Sheet_1.PDF]

**a**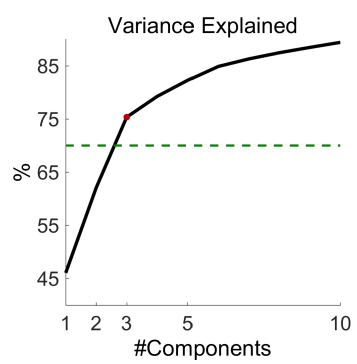**b**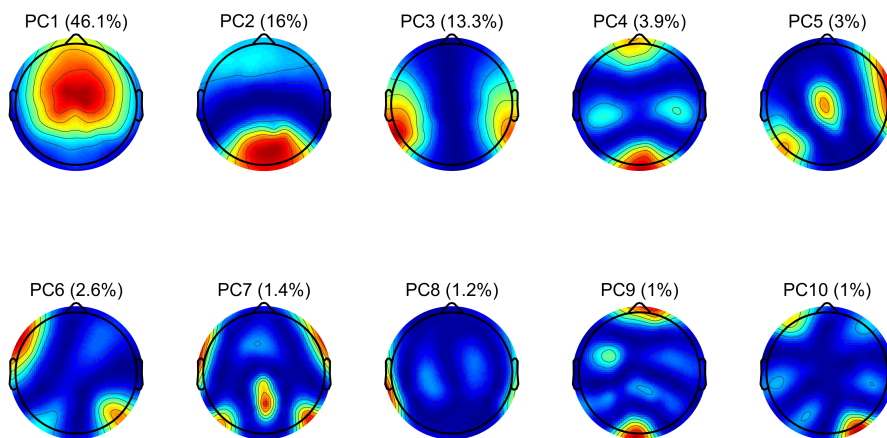

Supplement: Supplementary file 2 [file Data_Sheet_2.PDF]

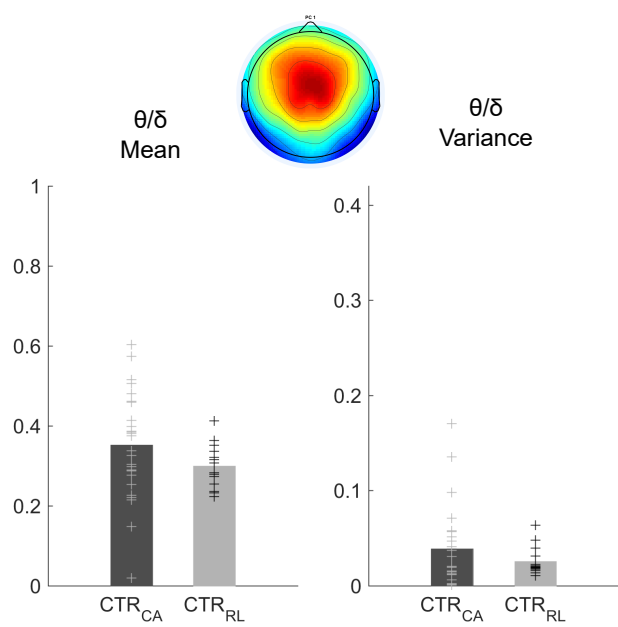

Supplement: Supplementary file 3 [file Data_Sheet_3.PDF]

**PC1**

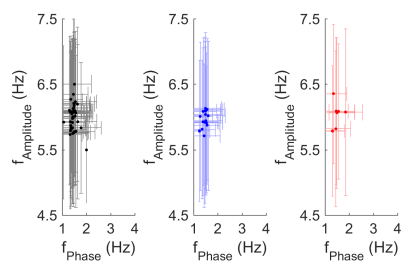

**PC2**

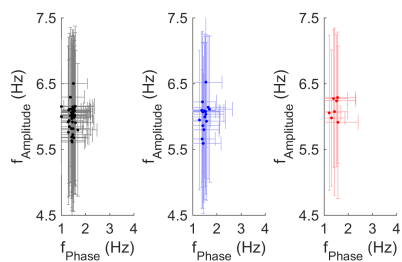

**PC3**

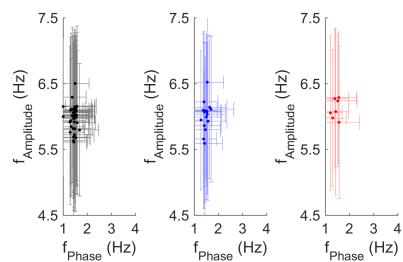

Supplement: Supplementary file 4 [file Data_Sheet_4.PDF]

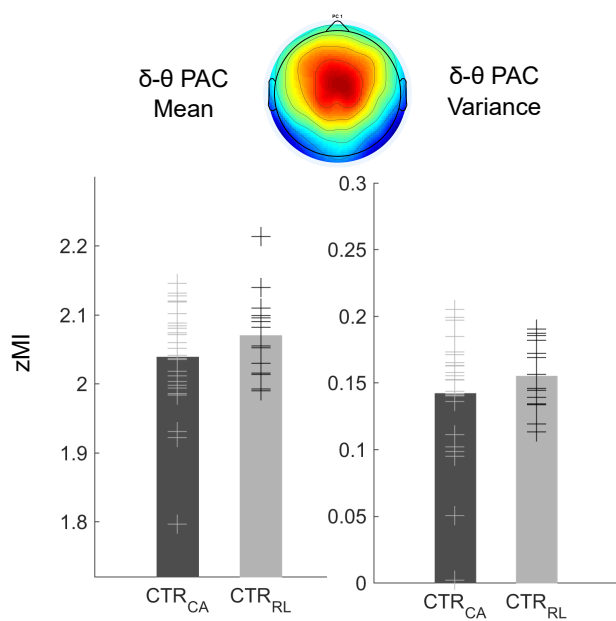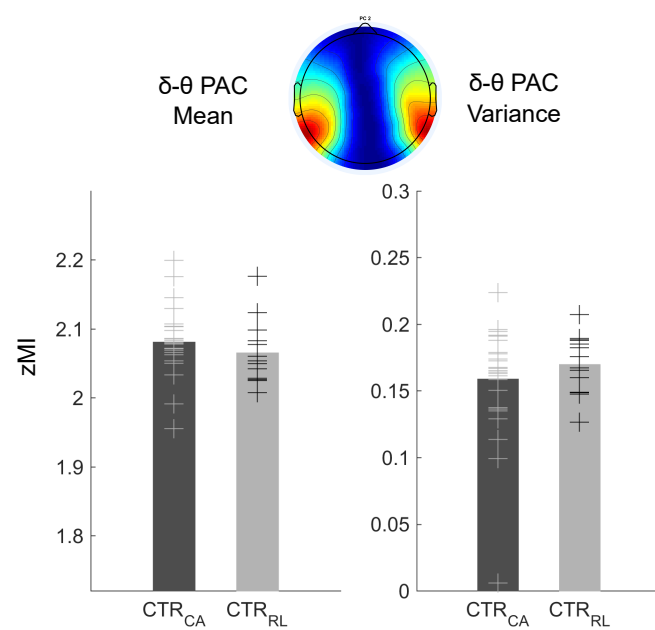

Supplement: Supplementary file 5 [file Data_Sheet_5.PDF]

**a**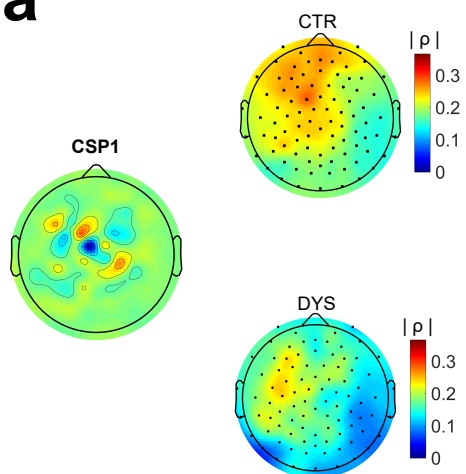**b**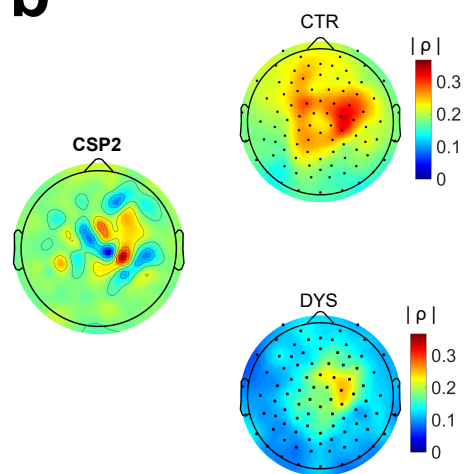**c**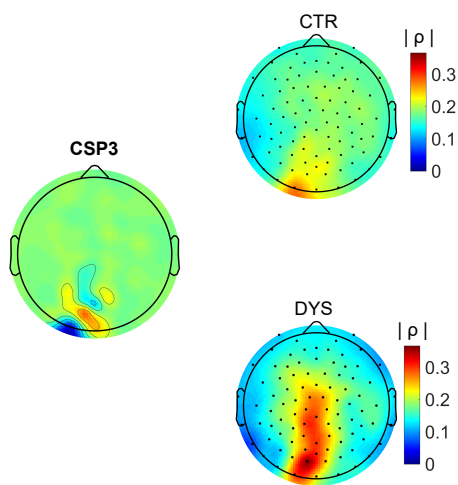**d**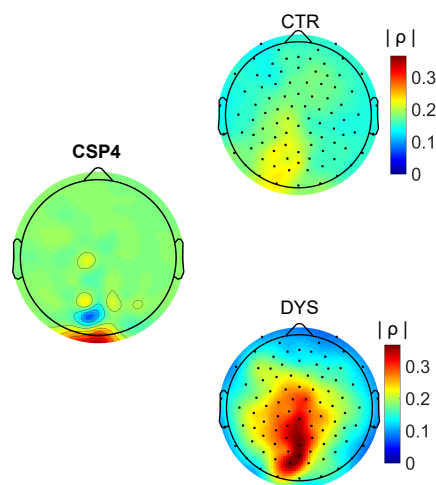

Supplement: Supplementary file 6 [file Data_Sheet_6.PDF]

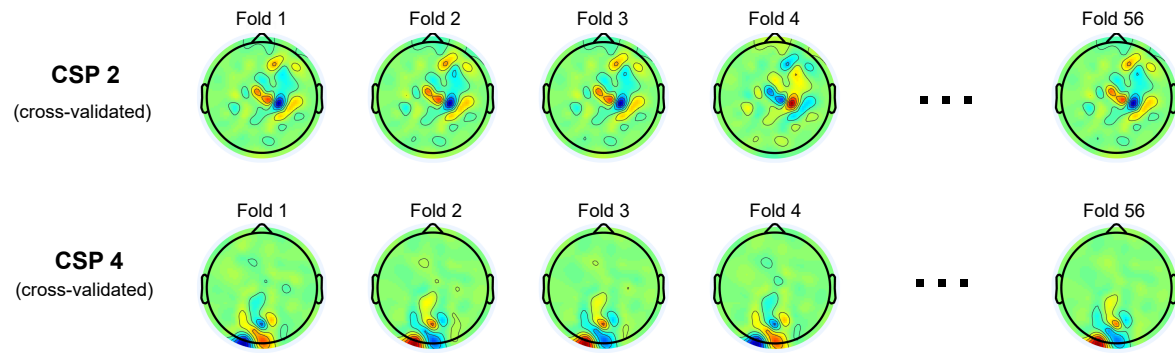

Supplement: Supplementary file 7 [file Data_Sheet_7.PDF]

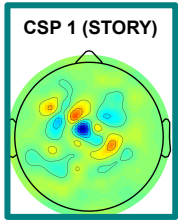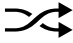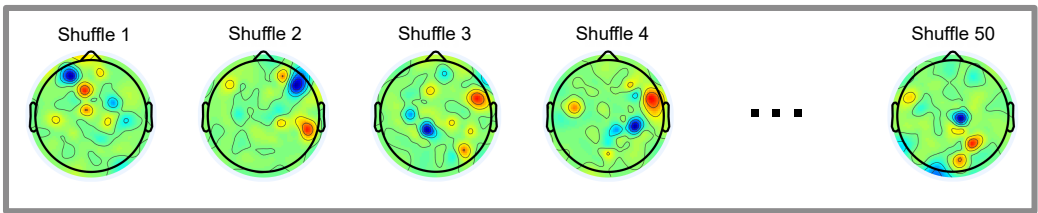

Supplement: Supplementary file 8 [file Data_Sheet_8.PDF]
