# Supplementary material for: Atypical low-frequency cortical encoding of speech identifies children with developmental dyslexia
Source: Front Hum Neurosci. 2024 Jun 7;18:1403677. doi: 10.3389/fnhum.2024.1403677 (PMC11190370; doi:10.3389/fnhum.2024.1403677)
Supplement: Supplementary file 9 [file Table_1.DOCX]

Supplemental Figure Captions

**Figure S1. PCA on the story listening EEG reveals importance of the 3 first components.**

(a) Percentage of variance explained using 1-10 PCs (see Methods on how Principal Components were calculated). PCs 1-3 explain over 70% of the total dataset variance (see also Figure 1b). From the 4^th^ component onwards, significantly lower variance percentages are explained.

(b) Channel weights for PCs 1-10 of the story listening EEG dataset. The sparse pattern of channel weights characterising components 4-10 suggest lower relevance of these components

**Figure S2. PCA on the rhythmic syllable EEG reveals importance of the 3 first components.**

(a) Percentage of variance explained using 1-10 PCs. PCs 1-3 explain over 70% of the total dataset variance (see also Figure 2b). Similarly to the story listening task, significantly lower variance percentages are explained from the 4^th^ component onwards.

(b) Channel weights for PCs 1-10 of the rhythmic syllable EEG dataset. In line with what happens with the story listening dataset, the sparse pattern of channel weights characterising components 4-10 suggest lower relevance for these components.

**Figure S3. Reading Level (RL) and Chronological Age (CA) control subgroupings from the typically-developing sample do not differ on theta/delta band ratio (mean or variance) for PC1** **during speech listening.**

As typically-developing children with a range of ages and reading levels participated in our study, it is possible that confounding factors like age and reading development affected their CSPs. Accordingly, we tested the robustness of the mean and variance of PC1 theta/delta ratio by splitting the typically-developing children into reading-level-matched (younger than dyslexics, similar reading level as dyslexics) and chronological-age-matched (same age as dyslexics, higher reading level) control groups. We then tested whether the groups differed in mean or variance of PC1 theta/delta power ratio. No differences between these subgroups were found (all uncorrected p-values > .13). Hence age and reading level do not affect the theta/delta band ratio in typically-developing children during story listening.

**Figure S4. High variance in the distribution of delta and theta frequencies of maximum cross-frequency coupling**.

(typically-developing children – black, dyslexic – blue, DLD – red).

**Figure S5.** **Reading Level (RL) and Chronological Age (CA) subgroupings of the typically-developing sample do not differ on delta-theta Phase-Amplitude Coupling (mean and variance) on both PCs 1 and 2**.

As typically-developing children with a range of ages and reading levels participated in our study, we also tested the robustness of delta-theta phase-amplitude coupling on the PCs that showed a significant group difference (see Figure 3b) for the age-matched and reading-level-matched subgroupings. As for theta-delta band ratio, no differences between subgroups were found (all uncorrected p-values > .1). Hence age and reading level do not affect the phase-amplitude coupling metric in typically-developing children during story listening.

**Figure S6. CSP filters are associated with distinct patterns of channel activity depending on group.**

(a,b) CSP filters that maximize signal variance for typically developing children (CTR) also show a higher correlation with larger anterior channel ensembles for this group – suggestive of a larger neurophysiological response. Dyslexics (DYS), on the other hand, show lower channel-CSP correlations and the higher correlations happen in smaller channel ensembles. Classifier features based on these filters are the ones with negative weights (see Figure 5c).

(c,d) The aforementioned pattern is reversed for CSP filters that maximize signal variance for dyslexic children with the DYS group showing higher correlation between these CSPs and large ensembles of channels in posterior regions of the scalp. Classifier features based on these filters are the ones with positive weights (see Figure 5c).

**Figure S7. Delta-CSP channel weights are highly stable over the cross-validation process during speech listening**

In our leave-one subject-out cross-validation (see Figure 5a and Methods), the training set covariance matrices of the dyslexic or control group used to calculate CSPs change for each fold, because we are holding test data from a different child on each fold. This may introduce an unpredictable source of variance on the CSPs. However, as depicted here for the cross-validated versions of CSPs 2 and 4, covariance changes appear to be minimal. Accordingly, delta-CSP weights remain fairly consistent throughout the cross-validation process.

**Figure S8. Depiction of the procedure for spatially redistributing the CSP weights to create shuffled versions of the story listening CSPs.**

Shuffled versions (long grey box) of the story listening CSP (teal box). This shuffling process was employed to investigate the importance of the CSP weight locations learned from the story listening dataset in classifying dyslexia status from the rhythmic syllable dataset. ROCs from the linear classifiers using the shuffled CSPs from the story listening task (for classification of the rhythmic syllable dataset) are depicted in grey in the main manuscript.
